# Shikonin Inhibits Cancer Through P21 Upregulation and Apoptosis Induction

**DOI:** 10.3389/fphar.2020.00861

**Published:** 2020-06-09

**Authors:** Fangfang Wang, Franklin Mayca Pozo, Danmei Tian, Xinran Geng, Xinsheng Yao, Youwei Zhang, Jinshan Tang

**Affiliations:** ^1^Institute of Traditional Chinese Medicine and Natural Products, Guangdong Province Key Laboratory of Pharmacodynamic Constituents of Traditional Chinese Medicine and New Drug Research, College of Pharmacy, Jinan University, Guangzhou, China; ^2^International Cooperative Laboratory of Traditional Chinese Medicine Modernization and Innovative Drug Development of Chinese Ministry of Education (MOE), College of Pharmacy, Jinan University, Guangzhou, China; ^3^Department of Pharmacology, Case Comprehensive Cancer Center, Case Western Reserve University School of Medicine, Cleveland, OH, United States

**Keywords:** Shikonin, anticancer effect, cell cycle arrest, autophagy, apoptosis, P21

## Abstract

Shikonin is a natural naphthoquinone compound and has demonstrated potent anti-cancer activities; however, the underlying molecular mechanisms remained elusive. Here we report that Shikonin inhibited the growth of a wide range of human cancer cell lines, illustrating a broad anticancer effect. Mechanistically, we show that Shikonin arrested the cell cycle at the G2/M phase, inhibited the ERK-dependent cell growth signal, and induced cell death in both P53 wild type and mutant cancer cells, which collectively contributed to the growth inhibitory effect of Shikonin. A pan-apoptosis inhibitor largely suppressed Shikonin-induced cell death, suggesting an important role of apoptosis in this process. Intriguingly, Shikonin also activated autophagy and inhibition of autophagy by depleting critical autophagic genes further increased Shikonin-induced cell death, indicating a protective role of autophagy. In uncovering the molecular mechanisms underlying these effects of Shikonin, we found that Shikonin induced a robust upregulation of P21 independent of the P53 status, upregulated autophagy genes, as well as inhibited expression of genes required for cell growth. Using mouse tumor models, we confirmed the strong anticancer effect of Shikonin *in vivo*. Together, our data reveal a broad range of pharmacological functions of Shikonin, involving simultaneous growth inhibition, cell cycle arrest, autophagy activation and apoptosis induction through regulating expression of critical genes involved in these pathways. Our study may facilitate the development of Shikonin in cancer therapy as a single agent or in combination with other anticancer therapies.

## Introduction

Zicao (purple gromwell), the dried root of *Lithospermum erythrorhizon* Sieb et Zucc, *Arnebia euchroma* (Royle) Johnst, or *Arnebia guttata* Bunge, is an herbal medicine that has been used to treat many kinds of illnesses in China and other Asian and European countries for centuries. A large number of studies have reported a wide range of biological activities of Zicao extracts including anti-inflammation, anti-oxidative stress, anti-virus, anti-bacteria and anti-cancer in both cultured cells and in animal models (Papageorgiou et al., 1999; Chen et al., 2002; Andujar et al., 2013; Wang et al., 2019). Shikonin is a major component of Zicao and belongs to the naphthoquinone family compound. Consistent with the reported function of Zicao extracts, Shikonin has demonstrated a broad spectrum of bioactivities including wound healing (Mani et al., 2004), anti-inflammation (Tanaka et al., 1986), anti-HIV (Chen et al., 2003), anti-cancer (Sankawa et al., 1977), and so on. It appears that its toxicity to normal tissues and organs is limited; hence, Shikonin has been extensively studied as an anti-cancer agent and had demonstrated promising effects both *in vitro* and *in vivo* (Papageorgiou et al., 1999; Chen et al., 2002; Andujar et al., 2013; Wang et al., 2019).

The molecular mechanisms underlying the anti-cancer activity of Shikonin seemed to be complicated and may depend on the cellular context (Wang et al., 2019). So far, the reported cellular targets of Shikonin include the pyruvate kinase isoenzyme M2 (PKM2) (Chen et al., 2011; Lu et al., 2018; Tang et al., 2018b), the MAPK pathway (Mao et al., 2008; Zhao et al., 2015; Shan et al., 2017), HIF1α (Li et al., 2017; Han et al., 2018; Tang et al., 2018b), JNK (Zhai et al., 2017; Lin et al., 2018), PI3K/AKT (Zhang et al., 2015; Zhou et al., 2017; Ni et al., 2018; Tang et al., 2018b), STAT3 (Qiu et al., 2017; Tang et al., 2018a), p16INK4A and p73 (Jang et al., 2015), and PTEN (Nigorikawa et al., 2006; Chen et al., 2018; Zhang et al., 2018). These findings, at one hand, demonstrate that Shikonin can regulate various biological processes (Wang et al., 2019). On the other hand, they also illustrate a conundrum as to how exactly Shikonin regulates cellular processes and how such regulation contributes to the anticancer activity of Shikonin.

In order to understand how Shikonin elicits its anti-cancer activity, in the current study, we systematically investigated the effect of Shikonin on both the short-term proliferation and the long-term survival of various cancer cell lines originated from lung, breast, pancreas, colon and bone and one normal cell line derived from the liver. We use both chemical and genetic approaches to determine the involvement of cellular processes such as cell cycle, autophagy and apoptosis in the anti-cancer effect of Shikonin. Our data reveal that Shikonin simultaneously induces cell cycle arrest, cell death and autophagy, which collectively control cancer cell growth, survival and death.

## Methods

### Chemicals, Cell Culture and Reagents

Shikonin (>99.0%, #517-89-5) and Z-VAD-FMK (#S7023) were from Selleck Chemicals (Huston, TX, USA). Rapamycin (#D9542) was purchased from Sigma (St. Louis, MO, USA). PEG300 (#P815612) and Tween 80 (#T818928) were purchased from Macklin Biochemical Co (Shanghai, China) or Selleck Chemicals (#S6704 and S6702, respectively). Propidium iodide (PI, # ICN19545810) was purchased from MP Biomedicals (Solon, OH, USA). Cell lines were grown in DMEM (U2OS, PANC-1 and MDA-MB-231), RPMI-1640 (A549), or Ham’s F12K medium (LO2) with 10% FBS (ExCell bio, China) and 1% penicillin–streptomycin (Gibco/ThermoFisher, Franklin, MA, USA) at 37°C in 5% CO_2_ and 98% humidity.

### Antibodies

Antibodies for β-Actin (#4970), p-mTOR (Ser-2448, #5536), p-ERK1/2 (Thr-202/Tyr-204, #4370), PARP (#9542S), caspase-3 (#9665S), anti-ULK1 (#8054S), anti-pRSK (#9344), anti-pATK (#9275S), anti-BECN1 (#3495S), cleaved Caspase-3 (#9664S) and BAX (#2772S), ERK1/2 (#9102) and HRP-conjugated secondary anti-rabbit and anti-mouse antibodies (#7076 and #7074) were from Cell Signaling Technology (Beverly, MA, USA). Anti-P53 (#SC-6243), anti-UHRF1 (#SC-373750) and anti-P21 (#SC-397) were from Santa Cruz Biotechnology (Santa Cruz, CA, USA). The anti-human LC3B (#NB100-2220) and anti-P62 (#NBP1-42821) antibodies were obtained from Novus Biologicals (Littleton, CO, USA). Anti-ATPA3 (#ab2826) antibody was from Abcam (Cambridge, UK).

### Cell Proliferation and IC_50_ Determination

Cell proliferation was determined by the cell counting kit-8 (cck-8) assay (BeyotimeInst Biotech, China). Briefly, 5 × 10^3^ A549 cells were seeded in each well of 96-well plates, grown at 37°C for 24 h, and treated with different concentrations of Shikonin for 1, 2, 3 and 4 days. Then 10 μl of cck-8 solution was added to each well, incubated at 37°C for 2 h and the absorbance was determined at 570 nm from five replicates using a microplate reader (Synergy TM HT, BioTEK, USA).

To determine the IC_50_ of Shikonin, A549, PANC-1, MDA-MB-131 and U2OS cancer cells, as well as LO2 liver fibroblasts, were treated with increasing dosages of Shikonin for 48 h and cell viability was determined as above-mentioned. Absorption in the blank well was subtracted and that in the DMSO control was set as 100%, and others were normalized accordingly. The IC_50_ was calculated by the GraphPad Prism program.

### Cell Cycle Analysis

For cell cycle analysis, 5 × 10^5^ A549 or PANC-1 cells were seeded in 6-cm dishes, treated with Shikonin for 0, 12 and 24 h. Cells were harvested, washed once with cold PBS, fixed in 70% ethanol, washed with PBS once, re-suspended in 1 ml of propidium iodide (PI) staining solution (50 mg/ml PI and 1 mg/ml RNase in sodium citrate buffer, pH 7.4), incubated in the dark for 30 min, and the cell cycle was measured by flow FACSCantoII cytometer (BD Biosciences, San Jose, CA, USA). Quantitation was performed using Multi-cycle Software (ModFit software) to determine the percentage of G1, S, G2/M and sub-G1 phase cells.

### Western Blotting

Total cell lysates were harvested from A549, PANC-1, MDA-MB-131, SW620, HT-29 or U2OS cells in lysis buffer (BeyotimeInst Biotech, China). The protein concentration was determined by the Pierce^®^ BCA Protein Assay Kit (Pierce, #23225, ThermoFisher, Franklin, MA, USA). Equal amount of total proteins (~40 μg) were separated on 6–10–15% SDS-PAGE, transferred to PVDF membranes (#IPVH00010, Millipore/Sigma, Burlington, MA, USA), blocked with 5% skim milk, probed with primary antibodies overnight at 4°C, incubated with HRP-conjugated secondary antibodies for 1 h at room temperature, and imaged by the Tanon 5200 chemiluminescence imaging system (BioTanon, China).

### Annexin V Analysis

Annexin V assay was conducted by the Annexin V fluorescein isothiocyanate (FITC) Apoptosis Detection Kit (KeyGEN BioTECH, Nanjing, China) according to manufacturer’s instructions. Briefly, A549 cells were seeded in 6-well plates at a density of 1 * 10^6^ cells/well in RPMI 1640 medium. The cells were treated or co-treated with 5 μM Shikonin, 200 nM CPT or both for 24 h at 37°C in 5% CO_2_, collected and stained with Annexin V-FITC and propidium iodide (PI) in the dark at room temperature for 15 min and analyzed by flow cytometer (BD Biosciences, San Jose, CA, USA), and the results were analyzed using the software FACS Diva (BD Biosciences, San Jose, CA, USA).

### RNA Interference (RNAi)

To generate ULK1 or BECN1 stably depleted cells, A549 or HeLa cells were stably infected with lentivirus shRNA vectors targeting these two genes, respectively. In brief, 6 * 10^6^ HEK293T cells in 100 mm dish were transfected with virus packaging plasmid combinations containing 5 μg individual shRNA lentivirus vector for ULK1 or BECN1, 2 μg pMDL, 1 μg VSVG and 1 μg RSV-REV. After 48 h, the supernatant was collected, centrifuged at 450*g* for 5 min at room temperature, and added with fresh culture media (1:1 vol/vol) into pre-seeded A549 or HeLa cells in 6-well plates. Polybrene (final concentration at 4 μg/ml) was added into the culture media 4 h later and incubated for 48–72 h. The cells were then split into 100 mm dishes and added puromycin (2–4 μg/ml) to select stable cell clones. After around 10 days (or until the colonies formed), the cells were pooled and protein expression of ULK1 or BECN1 was confirmed by immunoblotting. shRNA vectors targeting ULK1 (#TRCN0000000835 and # TRCN0000000838) and BECN1 (#TRCN0000033549 and # TRCN0000033552) were obtained from Sigma (St. Louis, MO, USA).

### QPCR Analysis

Total RNA was extracted from A549 cell cultures by the RNeasy plus kit (#74134, Qiagen USA, Germany Town, MD, USA). The cDNA was synthesized using the Revert Aid first strand cDNA synthesis kit according to the manufacturer’s procedure (#K1622, ThermoFisher, Franklin, MA, USA). Real-time PCR was performed on a CFX96 Real-Time PCR system (Bio-Rad, Hercules, CA, USA) with SYBR Green Master Mix (#208054, Invitrogen/ThermoFisher, Franklin, MA, USA). The mRNA level of target gene was determined by analyzing 2^−ΔΔCt^ using HPRT1 as the internal control. The program used is: 95°C for 3 min, followed by 40 cycles of 95°C 10 s and 60°C 30 s. Immediately following the cycle, melt curve is determined by heating the sample to 95°C for 10 s, reducing to 60°C for 30 s, and then gradually increasing to 95°C with 0.5°C increment increase.

Primers for RT-PCR are:

TP53-F: 5′-CTTCCATTTGCTTTGTCCCG-3′
TP53-R: 5′-CATCTCCCAAACATCCCTCAC-3′
CDKN1A (P21)-F: 5′-AACTAGGCGGTTGAATGAGAG-3′
CDKN1A (P21)-R: 5′-GAGGAAGTAGCTGGCATGAAG-3′
c-FOS-F: 5′-CTCAAGTCCTTACCTCTTCCG-3′
c-FOS-R: 5′-GAGAAAAGAGACACAGACCCAG-3′
JUN-F: 5′-TGTCCGAGAACTAAAGCCAAG-3′
JUN-R: 5′-TCAATGTTAACGAAAAGTCCAACG-3′
ATG3-F: 5′-GATGGCGGATGGGTAGATACA-3′
ATG3-R: 5′-TCTTCACATAGTGCTGAGCAATC-3′
ATG5-F: 5′-AAAGATGTGCTTCGAGATGTGT-3′
ATG5-R: 5′-CACTTTGTCAGTTACCAACGTCA-3′
HPRT1-F: 5′-AGCTTGCTGGTGAAAAGGA-3′
HPRT1-R: 5′-CCAAACTCAACTTGAACTCTCATC-3′

### Clonogenic Cell Survival Assay

Clonogenic survival assay was used to determine the long-term survival capability of cells. Briefly, after treatment, 5000 A549, PANC-1, U2OS, MDA-MB-231 or LO2 cells were seeded into 6-well plates in triplicate and cultured in drug-free full media for 10–14 days or until cell colonies were clearly visible. The culture medium was changed every 3 days. Cells were washed once with phosphate buffered saline and fixed in acetic acid-methanol solution (1:7, *vol*/*vol*) at room temperature for 5 min. After staining with 0.1% crystal violet dye in methanol at room temperature for 15 min, the plates were then gently rinsed under tap water, placed upside down to allow air dry. The dried plates were first scanned, then dissolved in 1% SDS. The plates were placed on a shaker to incubate until no areas of dense coloration were visible at the bottom of wells. Finally, the absorbance of each well was read at 570 nM using a microplate reader (Synergy TM HT, BioTEK, USA).

### Mouse Studies

For mouse studies, 5–6 weeks old female BALB/c Nu/Nu mice were purchased from Beijing HFK Bioscience CO. LTD (Beijing, China) or the Jackson Laboratory (Bar Harbor, ME, USA). All mice were housed in-group in cages with bedding, controlled temperature (23 ± 2°C), humidity (50 ± 5%) and illumination (12 h light/dark cycle). Mice were adapted to the facility for 1 week before experiments. All animal experiments were performed in accordance with the National Institutes of Health’s Guide for the Care and Use of Laboratory Animals (NIH publication No. 80-23, revised in 1996) and were approved by the Institutional Animal Care and Use Committee at Jinan University or Case Western Reserve University. In addition to all procedures to be sterile, mice were allowed access to sterile food and water and libitum. Shikonin was prepared at 140 or 70 mM stock solution in DMSO. The injection solution was always freshly prepared by mixing the stock solution with PEG300 (30%), Tween-80 (5%) and sterile H_2_O (various based on the Shikonin concentration). The solution is stable for at least 4 hours at room temperature, which is sufficient for injection.

For animal toxicity experiment, mice were injected with 20, 10, 5, 3 and 1 mg/kg Shikonin i.p. twice a week for 2 weeks. Animal survival was documented and plotted by Kaplan–Meier survival curve. For xenografted tumor study, 5 × 10^6^ A549 cells suspended in RPMI-1640 medium without serum were injected subcutaneously into the right flank of each mouse. Tumors were allowed to grow till the volume reached approximately ~100 mm^3^, and mice were randomly divided into the following two groups with 6 mice in each group: (1) Control; and (2) Shikonin (2 mg/kg). Shikonin was given by i.p. twice a week. Tumor volume and body weight were measured at least twice a week for 3 weeks. Tumor volume was calculated using the formula V = (L × W^2^) × 0.52 where V = volume, L = length, W = width.

### Statistical Analysis

All cell culture experiments were performed at least twice. Data are presented as mean ± standard deviation. The statistical analysis was conducted by the Prism 5.0 software. Pairwise comparison was performed using a two-tailed Student *t*-test, whereas one-way ANOVA was used to compare multiple comparisons. *P*-values of less than at least 0.05 were considered statistically significant.

## Results

### Shikonin Displays a Broad Inhibitory Effect on Cancer Cell Growth

**Figure 1:**
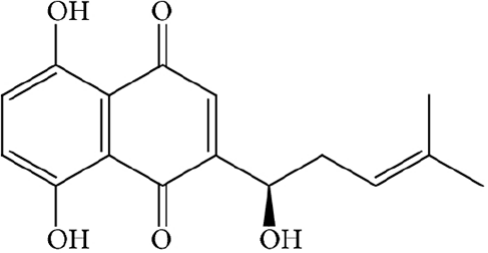
Chemical structure of Shikonin.

**Figure 2:**
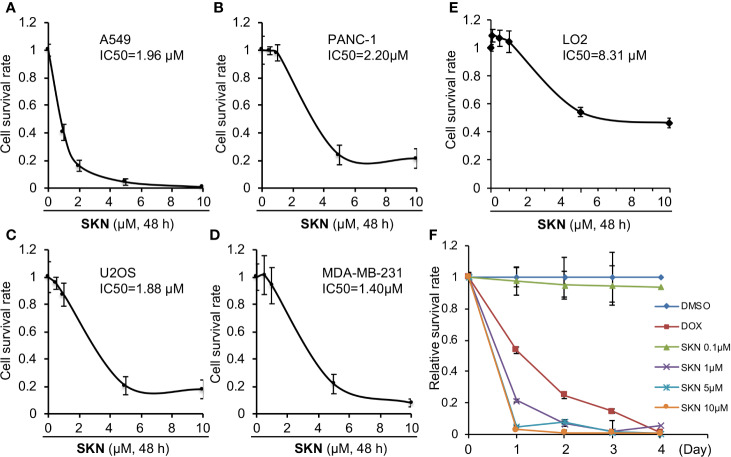
Effects of Shikonin on cancer cell growth. A549 **(A)**, PANC-1 **(B)**, U2OS **(C)**, MDA-MB-231 **(D)**, or LO2 **(E)** cells were treated with indicated concentrations of Shikonin for 48 h, and cell survival was analyzed by the CCK-8 assay. Data were normalized to that of DMSO control and presented as relative survival rate from five replicates. IC_50_ values were analyzed using the GraphPad software. **(E)** A549 cells were treated with DMSO or different concentrations of Shikonin or 10 μM doxorubicin (DOX) for 1, 2, 3 and 4 days, and cell survival was analyzed by the CCK-8 assay. The absorbance of Shikonin-treated group was normalized to that of the same day DMSO control, which yields the relative survival rate. Data represent mean and standard deviation from five replicates. **(F)** A549 cells were treated with DMSO or different concentrations of Shikonin for 1, 2, 3 and 4 days, and cell survival was analyzed by CCK-8 assay. The absorbance of Shikonin-treated group was normalized to that from the same day DMSO control, which yields the relative survival rate. Data represent mean and standard deviation from five replicates.

To understand the anti-cancer mechanisms of Shikonin (**Figure 1**), we first evaluated the IC_50_ of this compound in four different cancer cell lines including lung adenocarcinoma (A549), triple-negative breast cancer (MDA-MB-231), pancreatic cancer (PANC-1), and osteosarcoma (U2OS). We reason that an evaluation of the effect of Shikonin on these cancer cell lines may provide insights into the preclinical development of this agent in the future. The results show that Shikonin treatment for 48 h displayed an IC_50_ at around 1–2 μM in all cancer cell lines tested (**Figures 2A–D**). On the other hand, the IC_50_ of Shikonin in a normal human hepatocyte LO2 cell line is roughly 4-fold higher than that in cancer cells (**Figure 2E**). To further probe the growth inhibitory effect of Shikonin, we performed a real time cell proliferation assay using A549 as the representative cell line. The results show that Shikonin both dose- and time-dependently suppressed cancer cell proliferation (**Figure 2F**). At a dose at or greater than 1 μM, Shikonin showed even more growth inhibitory effect than a chemotherapeutic drug doxorubicin (**Figure 2F**), indicating a strong anti-cancer effect of Shikonin.

**Figure 3:**
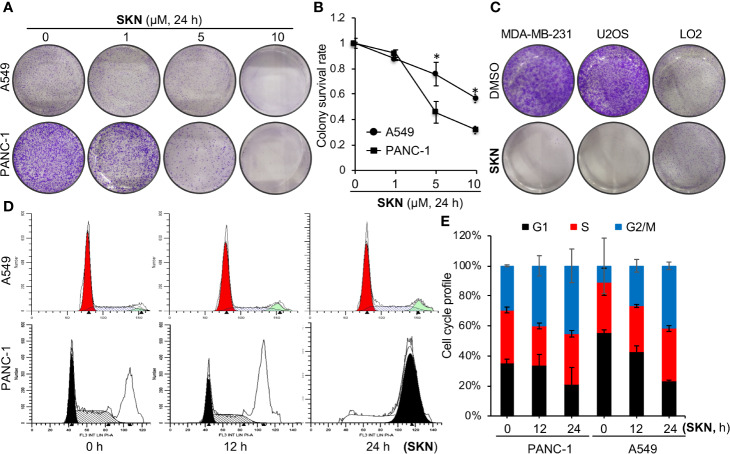
Effects of Shikonin on cell survival and cell cycle. **(A)** A549 or PANC-1 cells were treated with increasing concentrations of Shikonin for 24 h, and clonogenic survival assay was measured. Representative images are shown. **(B)** Quantitation of relative survival from **(A)**. Data represent mean and standard deviation from five replicates. *P <0.001 compared with the DMSO control. **(C)** MDA-MB-231, U2OS or LO2 cells were treated with 5 μM of Shikonin for 24 h, and clonogenic survival assay was measured. Representative images are shown. **(D)** A549 or PANC-1 cells were treated with 5 μM of Shikonin for 0, 12 and 24 h, fixed and cell cycle profile was analyzed. **(E)** Quantitation of cell cycle distribution for A549 and PANC-1 cells from ***D***. Data represent average and standard deviation from two independent experiments.

Then to determine the impact of Shikonin on long-term cell survival, we performed a dose-dependent clonogenic survival assay in A549 and PNAC-1 cancer cells. The results show that Shikonin dose-dependently inhibited the long-term survival of both cancer cell lines (**Figures 3A, B**). Similar inhibitory effects were observed in MDA-MD-231 and U2OS cancer cells (**Figure 3C**). On the other hand, Shikonin did not inhibit the long-term survival of the normal LO2 cell (**Figure 3C**), even though it also inhibited the short-term cell proliferation albeit at a much lesser degree than cancer cells (**Figure 2E**). Together, these data demonstrate a general anti-cancer effect of Shikonin and suggest that it has much less inhibitory effect on normal cells. These findings also allow us to choose selected cell lines from these four types to perform subsequent studies in order to avoid redundancy.

### Shikonin Induces G2/M Phase Cell Cycle Arrest and Robust Cell Death

To understand how Shikonin inhibited cancer cell growth, we first monitored the cell cycle profile in the presence or absence of Shikonin. Flow cytometry data revealed that Shikonin induced a time-dependent accumulation of cells at the G2/M phase in A549 and PANC-1 (**Figures 3D, E**) cells, suggesting that Shikonin induces cell cycle arrest at the G2/M phase.

**Figure 4:**
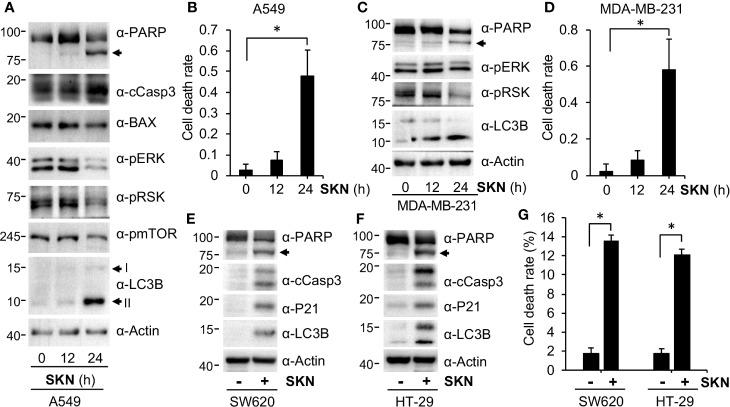
Shikonin induces cell death. **(A)** PANC-1 cells were treated with 5 μM of Shikonin for 0, 12 and 24 h, and protein expression was measured using specific antibodies. I and II indicate non-modified and lipidated forms of LC3B, respectively. Anti-PARP recognizes both full-length and cleaved PARP proteins, and the latter is indicated by the arrow. **(B)** Quantitation of dead cell population from cells in **(A)** by trypan blue staining. Data represent mean and standard deviation from five replicates. *P <0.001. **(C)** MDA-MD-231 cells were treated with 5 μM of Shikonin for 0, 12 and 24 h, and protein expression was measured by specific antibodies. **(D)** Quantitation of dead cell population from ***C*** by trypan blue staining. Data represent mean and standard deviation from five replicates. *P <0.001. SW620 **(E)** or HT-29 **(F)** cancer cells were treated with 5 μM of Shikonin for 12 h, and protein expression was measured by specific antibodies. **(G)** Quantitation of dead cell population from ***E*** and ***F*** by trypan blue staining. Data represent mean and standard deviation from five replicates. *P < 0.001.

To understand the molecular basis underlying the growth inhibitory effect of Shikonin, we conducted immunoblotting to examine expression of genes involved in cell growth, cell death and autophagy by Shikonin. We found that treatment of A549 cells with Shikonin time-dependently increased the cleavage of poly (ADP-ribose) polymerase (PARP) and caspase 3 (cCasp3) (**Figure 4A**), two known apoptotic cell death markers (Fernandes-Alnemri et al., 1994; Nicholson et al., 1995; Duriez and Shah, 1997). These findings are in general agreement with previous reports (review (Wang et al., 2019)). To confirm that Shikonin indeed induced cell death, we measured dead cell population using trypan blue staining. The results show that Shikonin induced significant cell death induction after 24 h treatment (**Figure 4B**). Similar results were obtained from breast MDA-MB-231 cancer cells (**Figures 4C, D**) and colorectal SW620 and HT-29 cancer cells (**Figures 4E–G**). Except A549, PANC-1, MDA-MB-231, SW620 and HT-29 are all P53 mutant cancer cell line. MDA-MB-231 carries a homozygous c.839G > A (R280K) mutation, whereas PANC1 is homozygous for c.818G > A (R273H). On the other hand, SW620 carries both R273H and P309S mutations whereas HT-29 is a R273H P53 mutant cancer cell line. Hence, these results demonstrate that Shikonin induces cell death in both P53 wild type and mutant cancer cell lines.

### Shikonin Inhibits the ERK Growth Signal Pathway

Shikonin had been previously reported to both inhibit and activate the extracellular signal-regulated kinas (ERK) pathway, providing inconsistent results of Shikonin (review (Wang et al., 2019)). During analyses, we observed that Shikonin time-dependently inhibited phosphorylation of ERK in both A549 and MDA-MB-231 cells (**Figures 4A, C**), indicating that Shikonin inhibited the ERK signaling. In agreement with the inhibition in phosphorylation of ERK, phosphorylation of the ERK downstream factor RSK was also reduced by Shikonin in a time-dependent manner (**Figures 4A, C**). These results suggest that Shikonin inhibits, but not activates, the ERK pathway, which may lead to the cell growth inhibitory effect of Shikonin.

### Shikonin Mainly Induces Apoptotic Cell Death

**Figure 5:**
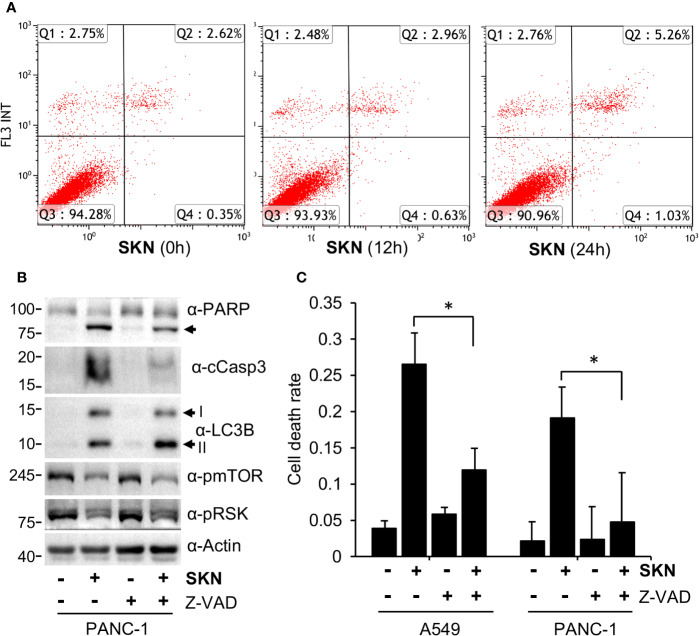
Apoptosis-dependent cell death induction by Shikonin. **(A)** A549 cells were treated with 5 μM of Shikonin for 0, 12 and 24 h, and apoptotic cells were analyzed by Annexin V staining. **(B)** PANC-1 cells were treated with 5 μM of Shikonin in the presence or absence of 10 μM Z-VAD for 24 h, and protein expression was measured by specific antibodies. Cleaved PARP, LC3B forms I and II are indicated by arrows. **(C)** Quantitation of dead cell population from ***B*** by trypan blue staining. Data represent mean and standard deviation from five replicates. *P < 0.001.

To confirm the role of apoptosis in Shikonin-induced cell death, we first carried out Annexin V staining. The FACS results show that Shikonin time-dependently induced a moderate increase in the apoptotic cell population (**Figure 5A**), suggesting the activation of apoptosis by Shikonin. However, Annexin V staining illustrated a much weaker activity of Shikonin than that of PARP cleavage or trypan blue staining. Such a relatively weak effect of Annexin V might be due to the insensitivity of the assay in measuring dead cells in this particular experimental setting, as well as the involvement of other cell death mechanisms.

To answer this question and to confirm the involvement of apoptosis, we assessed the effect of a pan apoptosis inhibitor, Z-VAD-FMK (Z-VAD), on Shikonin-induced cell death. The results show that Z-VAD co-treatment dramatically reduced the levels of cleaved PARP or Caspase 3 induced by Shikonin (**Figure 5B**). Consistently, Z-VAD significantly reduced the dead cell population of both A549 and PNAC-1 cells measured by trypan blue staining in the presence of Shikonin (**Figure 5C**). We realize that the effect of Z-VAD was weaker in PANC-1 cells than in A549 cells and that it did not completely block Shikonin-induced cell death (**Figure 5C**), suggesting that other types of cell death such as necroptosis might also be involved (review (Wang et al., 2019)).

### Shikonin Activates the Protective Autophagy Pathway

**Figure 6:**
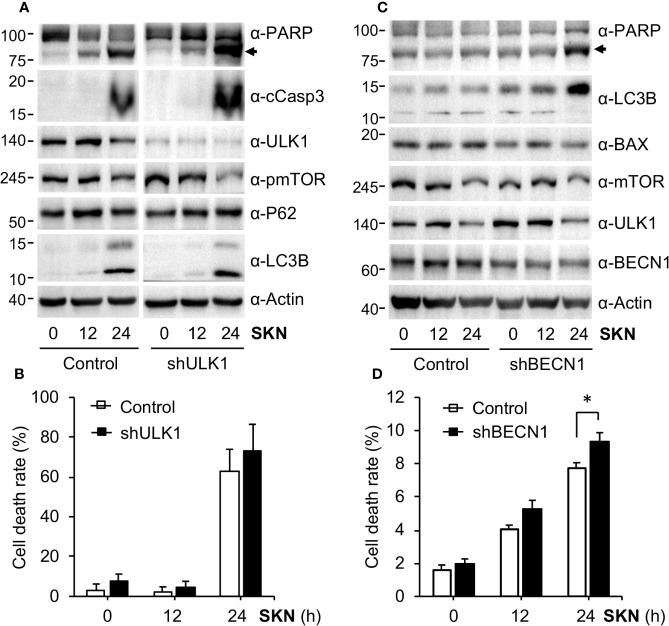
Shikonin activated autophagy. **(A)** A549 control or ULK1 depleted cells were treated with 5 μM of Shikonin for 0, 12 and 24 h, and protein expression was measured. **(B)** Quantitation of dead cell population from **(A)** by trypan blue staining. Data represent mean and standard deviation from five replicates. **(C)** HeLa control or BECN1 stably depleted cells were treated with 5 μM of Shikonin for 0, 12 and 24 h, and protein expression was measured using specific antibodies. **(D)** Quantitation of dead cell population from ***C*** by trypan blue staining. Data represent mean and standard deviation from five replicates. *P < 0.05.

Cells often activate the protective autophagy pathway to survive when encountering stressful situations, for instance, in the absence of nutrient or energy supply (Kundu and Thompson, 2008; Levine and Kroemer, 2008). Consistent with this idea and previous reports (review (Wang et al., 2019)), we found that Shikonin greatly increased the level of the lipidated form of LC3B (*i.e.*, LC3B II in **Figures 4A, B**), a well-known marker for autophagy activation (Kundu and Thompson, 2008; Mizushima and Komatsu, 2011). These results led us to further determine the role of autophagy in Shikonin-induced cellular response. To this end, we decided to inhibit autophagy by depleting critical autophagic genes including Unc-51 like autophagy activating kinase 1 (ULK1) and Beclin 1 (BECN1) by RNA interference (RNAi). ULK1 is a serine/threonine protein kinase that plays a critical role in the initiating step of autophagy (Matsuura et al., 1997; Kamada et al., 2000; Wirth et al., 2013). We generated an A549 cell line with ULK1 stably reduced (**Figure 6A**). We found that inhibiting ULK1 expression further increased the levels of cleaved Caspase 3 and PARP caused by Shikonin (**Figure 6A**), as well as elevated Shikonin-induced cell death (**Figure 6B**). BECN1, by forming a complex with the class III type phosphatidylinositol 3 kinase VPS34, is another key autophagy initiator (Funderburk et al., 2010). Therefore, we stably depleted BECN1 in HeLa cells and observed that even a partial depletion of BECN1 further elevated the level of cleaved PARP (**Figure 6C**) and cell death by Shikonin (**Figure 6D**), reinforcing the idea that autophagy is activated during Shikonin treatment. We noticed that Shikonin did not clearly induced LC3B lipidation in HeLa cells (**Figure 6C**). The reason is unclear at this moment; however, this could be a cell line specific effect as we constantly observed the conversion of LC3B in A549, PNAC-1, MDA-MB-231, SW620 and HT-29 cells. Overall, these results suggest that Shikonin generally activates autophagy and such autophagy activation helps to counteract the cell death-inducing effect of Shikonin.

### Shikonin Regulates Expression of Genes Involved in Cell Growth, Autophagy and Cell Cycle

**Figure 7:**
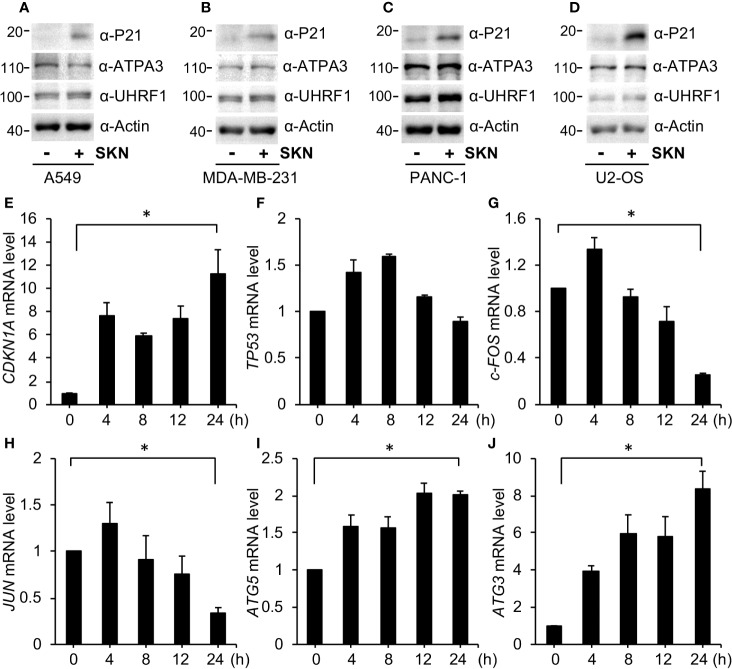
Shikonin upregulates P21 and alters gene transcription. **(A)** A549, **(B)** MDA-MB-231, **(C)** PANC-1 and **(D)** U2OS cells were treated with 5 μM of Shikonin for 6 h, and protein expression was measured by specific antibodies. For qPCR analysis, A549 cells were treated with 5 μM Shikonin for 0, 4, 8, 12 and 24 h, RNA was isolated, and expression of genes including *CDKN1A* **(E)**, *TP53* **(F)**, *c-FOS* **(G)**, *JUN* **(H)**, *ATG5* **(I)**, and *ATG3* **(J)** was analyzed. Data represent mean and standard deviation from five replicates. *P < 0.001 between 0 and 24 h groups.

Next we intended to further understand the molecular mechanisms by which Shikonin inhibited cell cycle progression, activated autophagy and induced cell death. We found that Shikonin treatment greatly increased the protein level of P21 in A549 (**Figure 7A**), MDA-MB-231 (**Figure 7B**), PNAC-1 (**Figure 7C**) and U2OS cells (**Figure 7D**), as well as in SW620 (**Figure 4E**) and HT-29 (**Figure 4F**) cells. Shikonin had also been previously reported to induce P21 expression in breast cancer cells (Zhang et al., 2013; Zheng et al., 2018). P21 is a potent inhibitor of cyclin-dependent kinases and induces cell cycle arrest in G1 or G2/M phase (Xiong et al., 1993; Abbas and Dutta, 2009; Karimian et al., 2016; Kastenhuber and Lowe, 2017). Hence, the upregulation in P21 is consistent with the G2/M phase cell cycle arrest by Shikonin. These data suggest that Shikonin induced upregulation in P21 in both P53 wild type and mutant cancer cell lines.

To confirm these results, we performed quantitative PCR (qPCR) to determine the mRNA level of *CDKN1A*, the gene encoding P21. The results show that Shikonin robustly increased *CDKN1A* levels in A549 cells in a time-dependent fashion (**Figure 7E**). On the other hand, Shikonin initially slightly increased *TP53* (the gene encoding P53) transcription followed by a reduction of it to the basal level (**Figure 7F**). The difference in transcription between *CDKN1A* and *TP53* again supports the idea that Shikonin-upregulated P21 is unrelated to P53, which is consistent with previous reports showing that P21 transcription can be both P53-dependent and -independent (Hoffman et al., 2002; Kastenhuber and Lowe, 2017).

To determine how Shikonin induced cell death and autophagy activation, we also assessed mRNA levels of critical genes involved in these pathways. We found that Shikonin time-dependently inhibited the expression of growth-promoting genes such as *JUN* and *c-FOS* (**Figures 7G, H**), as well as increased the levels of autophagy genes like *ATG3* and *ATG5* (**Figures 7I, J**).

### Shikonin Inhibits Tumor Growth in Mice

**Figure 8:**
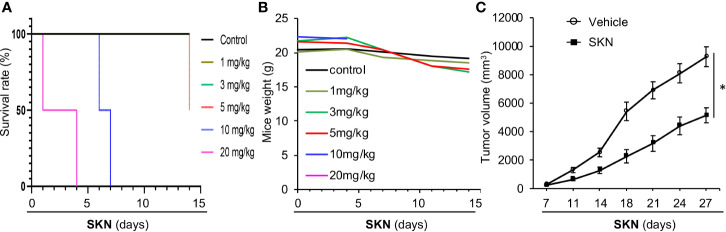
Mouse studies of Shikonin. **(A)** 5–6 week old nude mice (n = 2) were injected with vehicle control or 1, 3, 5, 10 or 20 mg/kg Shikonin i.p. twice a week for 2 weeks, and mouse survival was plotted by the Kaplen–Mier plotter. **(B)** Body weight of mice in **(A)** was recorded and plotted over time. **(C)** For nude mice tumor study, 5 * 10^6^ A549 cells were inoculated into nude mice subcutaneously. When the tumor reached around 100 mm^3^ in size, the mice were treated with 2 mg/kg of Shikonin i.p. twice a week for 3 weeks. Tumor volumes were measured during this time period. Data represent mean and standard error of tumor volume from six mice per group. *P < 0.001.

To determine if the strong *in vitro* anticancer effect of Shikonin can be recapitulated *in vivo*, we decided to perform mouse xenograft experiment. First, we assessed the toxicity of Shikonin in mice. We found that i.p. injection of Shikonin at a dose of 10 mg/kg and above caused acute death of mice, whereas a dose at 5 mg/kg induced partial death of the animal after 2 weeks of injection (**Figure 8A**). In contrast, a dose of 1–3 mg/kg did not result in mouse death (**Figure 8A**) despite slight weight loss of mice after 2 weeks of drug administration (**Figure 8B**).

Given the general anticancer effect of Shikonin observed in various cancer cell lines, we chose to use the A549 lung adenocarcinoma cell line as the representative to perform the *in vivo* study. To this end, we inoculated A549 cancer cells into nude mice subcutaneously, and examined the effect of Shikonin on tumor growth. Our data show that Shikonin significantly suppressed tumor growth in mice (**Figure 8C**), confirming the anticancer effect of this compound *in vivo*.

## Discussion

It has been shown that Shikonin elicits a wide variety of biological activities including the heavily studied anticancer effect (Wang et al., 2019). Yet, the underlying molecular mechanisms remained unclear. Studies have shown that Shikonin could inhibit cell proliferation, induce cell death and activate autophagy, pointing to complicated biological outcomes that often depend on the cellular context including cell types, treatment conditions and assay methods (Wang et al., 2019). In the current study, we presented evidence to show a broad anti-cancer effect of Shikonin that is largely consistent with previous reports. However, compared with previous reports, our studies are novel in a number of ways.

One of the major reasons for the uncertainty of the anti-cancer mechanism of Shikonin is that usually one type of cancer cell line was used and only short-term cell culture effect was measured using assays like MTT. Further, there is the lack of well-planned genetic and pharmacological studies to determine the time course of biological processes caused by Shikonin. Here, we systematically investigated the effect of Shikonin on both short-term and long-term growth of four different cancer cell lines and one normal cell line from the liver. In determining cell death (using trypan blue exclusion and Western blotting tools), two additional colorectal cancer cell lines were included to confirm the generality of Shikonin-induced cell death and autophagy activation. Our results reveal that Shikonin simultaneously inhibits cell cycle, induces cell death and activates autophagy. Although Shikonin had been reported to have effects on these biological processes, as far as we know, we are the first to show that Shikonin could do all of these at the same time in various cancer cell lines. These results clearly point out the complexity of the biological effect of Shikonin, as well as highlight a broad spectrum of activities of this compound.

Our studies advanced out understanding of cellular mechanisms underlying Shikonin-induced cell cycle arrest and cell death. Here we present strong evidence acquired from six different cancer cell lines from different tissues and organs to show that Shikonin arrested the cell cycle at the G2/M phase likely due to its up-regulation of P21, a potent cell cycle inhibitor that arrests cells at G1 or G2/M phase (Xiong et al., 1993; Abbas and Dutta, 2009; Karimian et al., 2016; Kastenhuber and Lowe, 2017). Such an increase in P21 was accompanied with elevated transcription. Interestingly, the increases in both P21 and cell death induced by Shikonin were observed in both P53 wild type and mutant cancer cell lines, highly supporting P53 status-independent activities of Shikonin.

Our studies also helped to clear some confusion about Shikonin in the literature. First, autophagy activation had been presented as both a positive and a negative factor for Shikonin-induced cell death. For instance, Kim et al. reported that autophagy activation protected cells from Shikonin-induced necroptosis (Kim et al., 2017), whereas another study suggested that autophagy activation contributed to Shikonin-induced cell death (Zhu et al., 2019). Here we also confirmed autophagy activation by Shikonin in a wide range of cancer cell lines except HeLa. We further showed that autophagy protected, but not promoted, Shikonin-induced cell death, illustrating a protective effect of autophagy in the presence of Shikonin. Nonetheless, the effect of autophagy activator or autophagic gene depletion was generally weak, indicating that autophagy activation in the presence of Shikonin may merely represent a consequence of cellular reaction to stress (in this case, Shikonin treatment)(Wang et al., 2019). Second, the ERK kinase belongs to the MAPK family kinase and plays a critical role in regulating cell growth (Peti and Page, 2013). Not surprisingly, Shikonin has been shown to inhibit the ERK kinase, contributing to its growth inhibitory effect. However, a number of studies also reported that Shikonin activated ERK (Wu et al., 2005; Chang et al., 2010; Shen et al., 2012; Han et al., 2019) and such activation seemed to promote the cell death inducing effect of Shikonin (Zhuang and Schnellmann, 2006). Therefore, the effect of Shikonin on the ERK pathway remained controversial. Here, we show that Shikonin reduced the levels of activated ERK and its downstream factor RSK in multiple cancer cell lines, indicating that Shikonin inhibits the ERK pathway at least under these particular experimental conditions. qPCR results showed a reduction in the expression levels of growth-related genes, which is consistent with the data showing the inhibition of the ERK pathway. These results may help to clear the confusion as to the role of the ERK signal in the effect of Shikonin.

Also we, for the first time, provided a detailed toxicity analysis of Shikonin in nude mice. Our studies reveal a relatively low dose tolerance of Shikonin in mice (< 3 mg/kg). As to why previous studies reported even greater than 10 mg/kg dose of Shikonin in mice, we speculate that it was largely due to inappropriate solution making of Shikonin in previous studies. We have tested various solvents to allow stable dissolving of Shikonin. We found that the Shikonin solution can be stable for as long as 4 h at room temperature only when dissolved in PEG300 (30%), Tween-80 (5%) and sterile H_2_O. Otherwise, Shikonin seemed to be dissolved initially but will precipitate within 30 min, preventing the injection of precise amount of chemicals into mince. Consequently, that led to the incorrect impression that mice can tolerate high dose of Shikonin. This additional analysis allowed us to precisely control the amount of Shikonin and determine its toxicity in mice. By combining with the effect on normal cell line, we believe that we can lower the dose of Shikonin in future studies. Together, our results demonstrate strong anticancer effect of Shikonin across a wide range of human cancer cell lines and reveal activation of biological pathways that contributed to the growth inhibitory effect of Shikonin. These results will shed significant light on our understanding of this molecule in cancer therapy.

## Data Availability Statement

The raw data supporting the conclusions of this article will be made available by the authors, upon reasonable request.

## Ethics Statement

The animal study was reviewed and approved by Institutional Animal Care and Use Committee at Jinan University or Case Western Reserve University.

## Author Contributions

JT, XY, and YZ conceived the study, designed the experiments and wrote the manuscript with the help of co-authors. FW conducted all the majority of experiments and facilitated data analysis. FP and XG carried out the xenograft tumor study. All authors contributed to the article and approved the submitted version.

## Funding

JT is supported by National Science Foundation of China (Nos. 81728022, 81673320) and YZ is supported in part (30%) by NCI (CA230453).

## Conflict of Interest

The authors declare that the research was conducted in the absence of any commercial or financial relationships that could be construed as a potential conflict of interest.
