# Corrigendum: Shikonin Inhibits Cancer Through P21 Upregulation and Apoptosis Induction

**DOI:** 10.3389/fphar.2020.01088

**Published:** 2020-07-16

**Authors:** Fangfang Wang, Franklin Mayca Pozo, Danmei Tian, Xinran Geng, Xinsheng Yao, Youwei Zhang, Jinshan Tang

**Affiliations:** ^1^ Institute of Traditional Chinese Medicine and Natural Products, Guangdong Province Key Laboratory of Pharmacodynamic Constituents of Traditional Chinese Medicine and New Drug Research, College of Pharmacy, Jinan University, Guangzhou, China; ^2^ International Cooperative Laboratory of Traditional Chinese Medicine Modernization and Innovative Drug Development of Chinese Ministry of Education (MOE), College of Pharmacy, Jinan University, Guangzhou, China; ^3^ Department of Pharmacology, Case Comprehensive Cancer Center, Case Western Reserve University School of Medicine, Cleveland, OH, United States

**Keywords:** Shikonin, anticancer effect, cell cycle arrest, autophagy, apoptosis, P21

In the published article, there was an error regarding the contribution of “Xinsheng Yao,” who should be a co-corresponding author.

The authors apologize for this error and state that this does not change the scientific conclusions of the article in any way. The original article has been updated.

