# Supplementary figures and images for: Shikonin Inhibits Cancer Through P21 Upregulation and Apoptosis Induction
Source: Front Pharmacol. 2020 Jun 9;11:861. doi: 10.3389/fphar.2020.00861 (PMC7296065; doi:10.3389/fphar.2020.00861)

Figure 4C

Figure 4A

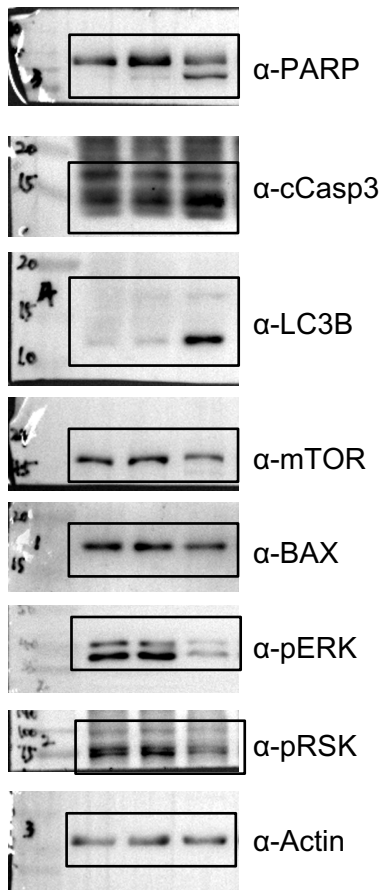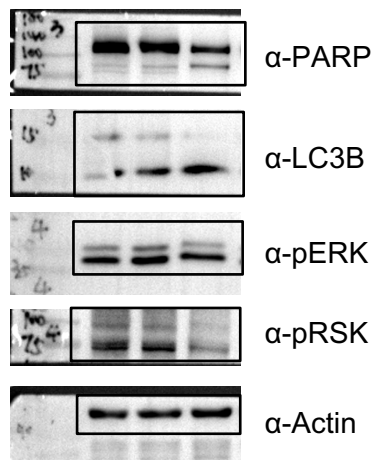

Figure 4F

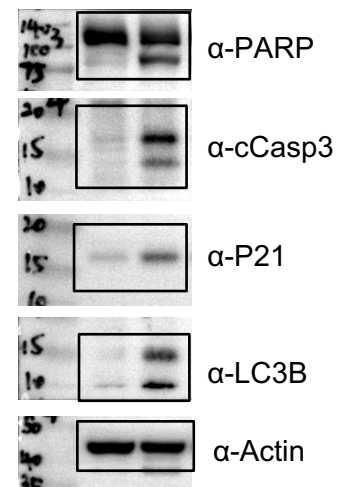

Figure 4E

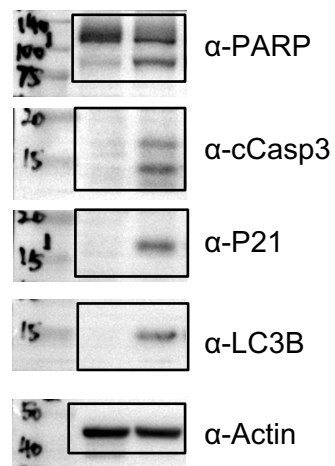

Figure 5B

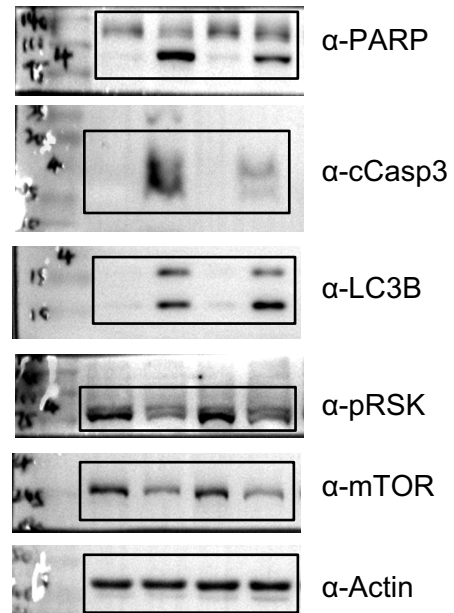

Figure 6A

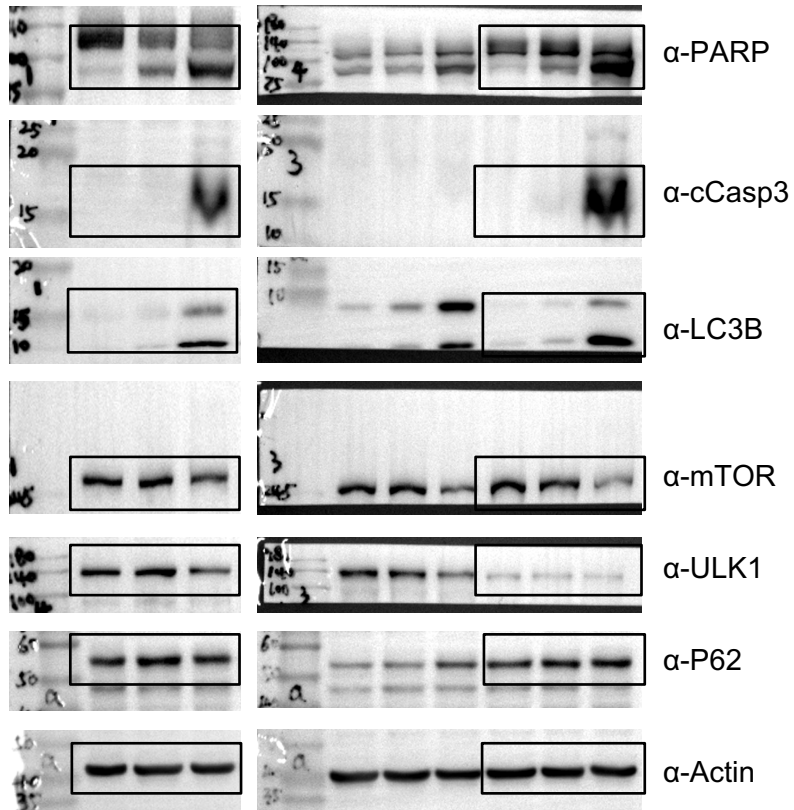

Figure 6C

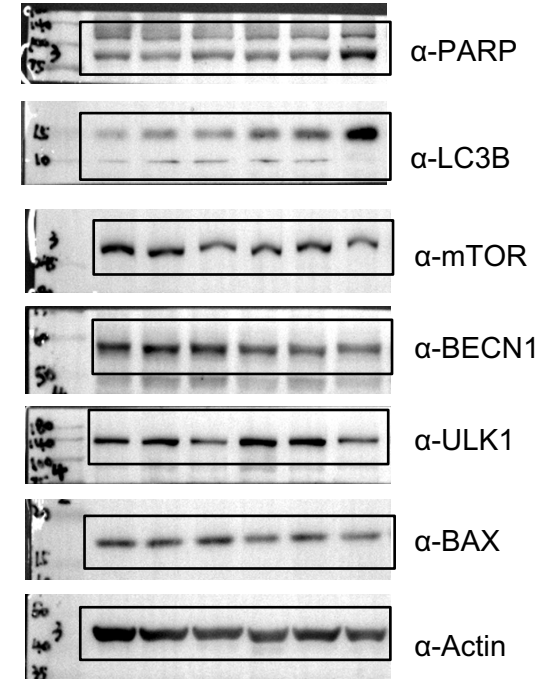

Figure 7A

Figure 7B

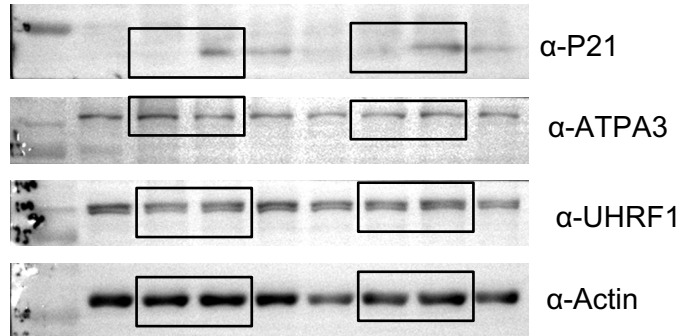

Figure 7C

Figure 7D

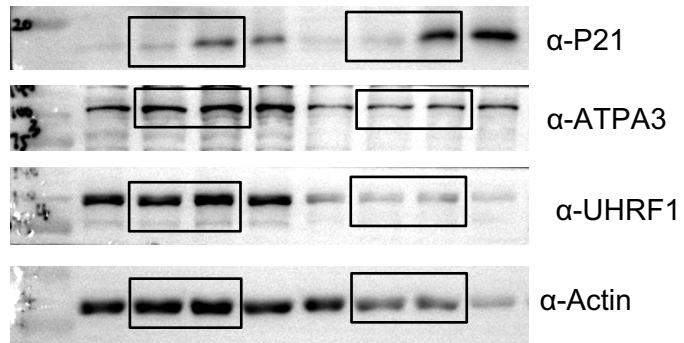

Supplement: Supplementary file 1 [file DataSheet_1.pdf]
